# Phylogenetic, population structure, and population demographic analyses reveal that *Vicia sepium* in Japan is native and not introduced

**DOI:** 10.1038/s41598-023-48079-4

**Published:** 2023-11-25

**Authors:** Ichiro Tamaki, Mizuo Mizuno, Tatsuo Ohtsuki, Kohtaroh Shutoh, Ryoichi Tabata, Yoshihiro Tsunamoto, Yoshihisa Suyama, Yusuke Nakajima, Naoki Kubo, Takeru Ito, Naohiko Noma, Emiko Harada

**Affiliations:** 1 Gifu Academy of Forest Science and Culture, 88 Sodai, Mino, Gifu 501-3714 Japan; 2 Gifu Prefectural Research Institute for Forests, 1128-1 Sodai, Mino, Gifu 501-3714 Japan; 3 Gifu Pharmaceutical University, 5-6-1 Mitahora-Higashi, Gifu, 502-8585 Japan; 4 Lake Biwa Museum, 1091 Oroshimo, Kusatsu, Shiga 525-0001 Japan; 5 The Hokkaido University Museum, Hokkaido University, Kita 10, Nishi 8, Kita-ku, Sapporo, Hokkaido 060-0810 Japan; 6 Research Institute of Energy, Environment and Geology, Hokkaido Research Organization, Kita 19 Nishi 12, Kita-ku, Sapporo, Hokkaido 060-0819 Japan; 7 Kawatabi Field Science Center, Graduate School of Agricultural Science, Tohoku University, 232-3 Yomogida, Naruko-Onsen, Osaki, Miyagi 989-6711 Japan; 8 School of Environmental Science, The University of Shiga Prefecture, 2500 Hassaka, Hikone, Shiga 522-8533 Japan

**Keywords:** Plant ecology, Plant evolution, Plant genetics, Plant molecular biology

## Abstract

*Vicia sepium* (bush vetch) is a perennial legume widely distributed throughout the Eurasian continent. However, its distribution in Japan is limited to Mt. Ibuki and small parts of central and southern Hokkaido. Therefore, each Japanese *V. sepium* lineage has been considered to have been introduced separately from Europe. Here, we examined whether the species was introduced or not on the basis of cpDNA sequences and genome-wide SNPs from Japanese and overseas samples. Both the cpDNA haplotype network and the nuclear DNA phylogenetic tree showed that Japanese *V. sepium* is monophyletic. Furthermore, although the nuclear DNA phylogenetic tree also showed that each lineage is clearly monophyletic, genetic admixture of the genetic cluster dominated in the Hokkaido lineage was also detected in the Mt. Ibuki lineage. Population divergence analysis showed that the two lineages diverged during the last glacial period. The Mt. Ibuki lineage showed a sudden population decline 300–400 years ago, indicating that some anthropogenic activity might be involved, while the Hokkaido lineage showed a gradual population decline from 5000 years ago. Consequently, these two lineages show low current genetic diversity compared with overseas lineages. These results show that the Japanese *V. sepium* is not introduced but is native.

## Introduction

*Vicia sepium* L. (bush vetch, Fabaceae) is a small perennial herb that is widely distributed on the Eurasian continent in the temperate Euro-Siberian broad-leaved forest region^1^. It has a wide distribution, both horizontally and vertically; for example, in the Swiss Alps, it grows from the lowlands to the sub alpine zone^2^. However, because its seed dispersal is autochory, the seed dispersal system is not suitable for long-distance dispersal over watersheds. It has been introduced by humans as a pasture plant for livestock and become naturalized in North America^1^. In Japan, it is reported from only a few regions: Mt. Ibuki, central Honshu; central and southern Hokkaido; and Mt. Ishizuchi, Shikoku. As the distribution areas in Japan are limited and distant from each other, it has been considered that the Japanese *V. sepium* was introduced from Europe and became naturalized there^3,4^.

Mt. Ibuki is a mountain in central Japan with a harsh endemic environment^5^. Although its altitude is not very high (1377 m), the winter snow depth is extreme, reaching 11.82 m in 1927, which is the highest record in the world. Its base soil consists of limestone and is characterized by good drainage and aridity, which limits the development of forests. This unique environment forms an endemic flora composed of plants adapted to the heavy snow, alpine plants and endemic species [e.g., *Veronica subsessilis* (Miq.) Carrière, *Cirsium confertissimum* Nakai, and *Euphrasia insignis* Wettst. subsp. *iinumae* (Takeda) T.Yamaz.]. These plants on Mt. Ibuki have been used by local people as medicine, fertilizer, or pasture plants since the seventeenth century^6^.

It is considered that the *V. sepium* on Mt. Ibuki originated from seeds accidentally mixed with seeds of medicinal plants brought from Europe in the sixteenth century by a European Jesuit missionary, when Nobunaga Oda, a regional governor at the time, directed the creation of a large medicinal plant garden of up to 50 ha^7–9^. However, no traces of this garden exist, and it is described only in an old document called “*Nanbanji-kohaiki*” (History of the rise and fall of Christian temples in Japan)^10^. Therefore, the existence of *V. sepium* and two other non-medicinal plants of putative European origin [*Lathyrus pratensis* L. and *Elymus caninus* (L.) L.] is taken as evidence for this garden. Another old document, called “*Somoku-zusetsu*” (Figures and descriptions of plants)^11^, reported that a number of *V. sepium* individuals were growing on Mt. Ibuki. Although this document names this plant “Karasunoendo”, one of the Japanese names for *Vicia sativa* L., the plant can be identified as *V. sepium* by the rounded shape of the leaf tip in the drawing. In addition, *V. sativa*, drawn with its characteristic concave leaf tip, is documented on another page of the book as “Yahazunoendo”. This evidence suggests that *V. sepium* grew on Mt. Ibuki at least 166 years ago.

In Hokkaido, *V. sepium* is recorded from one central and two southern localities. A number of specimens from around Naganuma-cho, central Hokkaido, are stored in the herbarium of the Hokkaido University Museum (SAPS), which is the most authoritative herbarium in Hokkaido. In this area, the species grows mainly in artificial meadows on riverbank slopes^12^ that are close to sites of human disturbance. SAPS holds two old specimens of *V. sepium* collected from central Hokkaido in 1878 and 1894 (*Class’80s.n.*, July 1878, SAPS066754 and *H. Nagaya s.n.*, early June 1894, SAPS066753), which suggest that *V. sepium* was already growing here at least 145 years ago. On the basis of this evidence, it has been assumed that *V. sepium* was introduced into Hokkaido in the early Meiji era (late nineteenth century), during the early colonization of Hokkaido, by contamination of pasture seeds^4,12^.

In southern Hokkaido, it is recorded from Samani-cho and Matsumae-cho. The former is reported only in Takahashi^13^, and no specimens are stored in SAPS. Therefore, its distribution in Samani-cho is uncertain. The latter is a recently found new site, and two specimens are held (*T. Shimazaki 21-023*, 5 May 2021, SAPS061611 and *T. Shimazaki 21-047*, 24 May 2021, SAPS061868). Their habitats are bedrock on a ridge and meadow along a trail, respectively. In aerial photographs in Google Maps (https://www.google.com/maps/, accessed on 13 April 2023), the new collection site seems to be a natural environment covered by forests and far from human disturbance. This new finding raises the question of whether *V. sepium* growing here is introduced or native.

*Vicia sepium* is also recorded from Mt. Ishizuchi, Shikoku^3,4^. These descriptions are probably based on the flora list of Mt. Ishizuchi reported by Jinno and Yamamoto^14^. Although “*V. sepium*” is listed, the Japanese name is given as “Karasunoendo”, one of the Japanese names for *V. sativa*. Since Jinno and Yamamoto^14^ did not illustrate the plants and no voucher specimens are recorded, unlike in the case of Mt. Ibuki, the plant may be *V. sativa* that was misidentified as *V. sepium*. In addition, *V. sepium* is not included in any of the later literature on the flora of Mt. Ishizuchi e.g.^15,16^. Thus, we considered that the distribution record of “*V. sepium*” on Mt. Ishizuchi indicates *V. sativa*.

**Figure 1:**
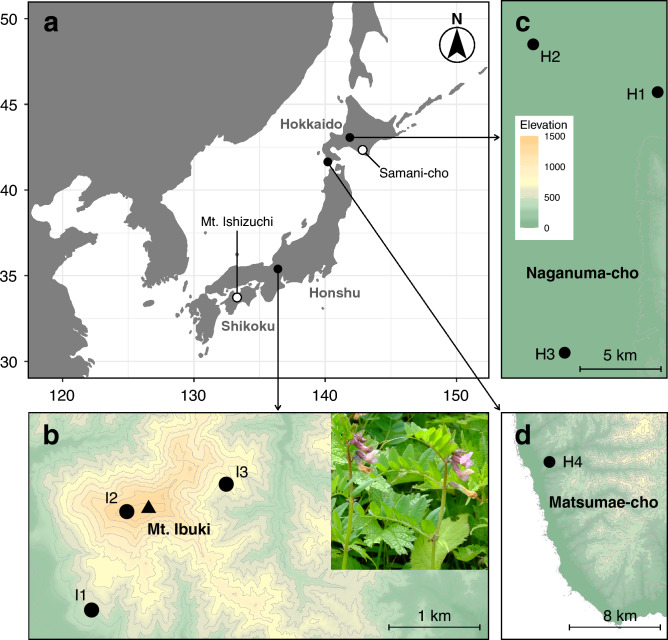
Distribution and locations of sampling sites of *Vicia sepium* in Japan. (**a**) Known distribution of *V. sepium* in Japan. ● Sample sites; ○ uncertain sites. (**b**–**d**) Population locations on (**b**) Mt. Ibuki, (**c**) central Hokkaido, (**d**) southern Hokkaido. Inset in (**b**) shows flowers of *V. sepium* in Mt. Ibuki. Maps were created by R packages ggplot2 version 3.4.2 (https://ggplot2.tidyverse.org) and maps version 3.4.1 (https://CRAN.R-project.org/package=maps) using digital elevation model data freely provided by the Geographical Information Authority of Japan (https://www.gsi.go.jp/).

Thus, only three regions in Japan are reliably known to have *V. sepium* growing: Mt. Ibuki and central and southern Hokkaido (Fig. 1). If it was introduced from Europe during the early modern period, there should be its signatures in its genome. When we estimate the trajectory of past population size changes of Japanese *V. sepium* populations over time, if only part of the diversity would have been introduced, the Japanese *V. sepium* population should show a sudden and severe population bottleneck at that time^17^. Furthermore, by comparing the phylogenetic relationships between Japanese and overseas samples, we may be able to get to the origin. Therefore, here, we investigated chloroplast and nuclear DNA data of Japanese and overseas (European, Siberian, and East Asian) *V. sepium* samples, and clarify the origin of Japanese *V. sepium* lineages, through phylogenetic, population structure, and population demographic analyses.

## Materials and methods

### Sampling and DNA extraction

We collected leaves of *V. sepium* from three populations in each on Mt. Ibuki and in central Hokkaido in 2016–2019 (Fig. 1). We also collected *V. sativa* and *Vicia hirsuta* as an outgroup in Mino, Japan, in 2016. Leaves were immediately dried with a silica gel and stored at room temperature until DNA extraction. DNA samples of German *V. sepium* plants were provided by the Botanic Garden and Botanical Museum Berlin-Dahlem^18^. We used leaves from southern Hokkaido and Russian *V. sepium* specimens stored in SAPS for DNA extraction. Details of the samples used in this study are summarized in Table 1. All plant samples were collected with permission. Genomic DNA was extracted from leaves by the CTAB method^19^ or with a DNeasy Plant Mini Kit (QIAGEN, Germany).

**Table 1:** Samples used in this study.

Species	Abbr.	Sample site	Latitude	Longitude	N_cp_	N_MIG_	MIG-seq library^a^
*V. sepium*	I1	Mt. Ibuki (Shiga Prefecture side), Japan	35.3970	136.3912	4	5	B
	I2	Mt. Ibuki (summit of the mountain), Japan	35.4190	136.4009	7	14	B (3) and T (11)
	I3	Mt. Ibuki (Gifu Prefecture side), Japan	35.4250	136.4279	6	48	T
	H1	Central Hokkaido (Iwamizawa-shi), Japan	43.0754	141.7446	5	3	B
	H2	Central Hokkaido (Nanporo-cho), Japan	43.1022	141.6472	4	3	B
	H3	Central Hokkaido (Naganuma-cho), Japan	42.9322	141.6709	3	4	B
	H4	Southern Hokkaido (Matsumae-cho), Japan^b^	41.5443	140.0195	3	0	–
	G	Germany (Soest, Forst, Kassel, Schleusingen and Frankfurt am Main)^c^	51.5303	8.1086	5	5	B
			51.7417	14.6278			
			51.2922	9.4636			
			50.4922	10.7753			
			50.1356	8.7194			
	R	Vladiovostok, Russia^d^	–	–	0	1	B
*V. sativa*	VSA	Mino, Gifu Prefecture, Japan	35.5559	136.9194	0	1	T
*V. hirsuta*	VHI	Mino, Gifu Prefecture, Japan	35.5559	136.9194	0	1	T

N_cp_ and N_MIG_, number of samples used in cpDNA sequencing and MIG-seq, respectively.

^a^Libraries adjusted at “B” Lake Biwa Museum and “T” Tohoku University. Numbers of samples in parentheses.

^b^The three DNA samples were extracted from specimens stored in the Hokkaido University Museum (SAPS). Although there were only two specimens, one of them contained two individuals and thus we could obtain three different DNA samples.

^c^Samples stored in the Botanic Garden and Botanical Museum Berlin-Dahlem.

^d^A sample stored in SAPS.

### Chloroplast DNA sequencing

Two cpDNA regions, *rbcL* and *matK*, were sequenced from 37 individuals (Table 1) with the primers listed in Table S1. Although we attempted to obtain sequences from a Russian sample, no DNA was amplified from either region. The total volume for the PCR was 5.5 μL, containing 1.0 μL of template DNA, 2.5 μL of SapphireAmp Fast PCR Master Mix (Takara Bio, Japan), and 0.2 μM of each primer. PCR was performed on a TaKaRa PCR Thermal Cycler Dice Touch (Takara Bio, Japan) with an initial denaturation for 1 min at 94 °C; 35 cycles of denaturation for 5 s at 95 °C, annealing for 5 s at 58 °C, and extension for 7 s at 72 °C; and a final extension for 5 min at 72 °C. After purification of the PCR products with ExoSAP-IT (Thermo Fisher Scientific, USA), sequencing and electrophoresis were performed at Hokkaido System Science, Japan, with a BigDye Terminator Cycle Sequencing Kit version 3.1 (Thermo Fisher Scientific, US). Sequence data were aligned with sequences obtained from GenBank (Table S2) in MEGA version 11 software^20^.

### High-throughput sequencing and SNP calling

Multiplexed inter-simple-sequence-repeat genotyping-by-sequencing (MIG-seq), which is effective for low-quality DNA such as that extracted from stored samples, was used to identify the genotypes of nuclear genomes^21^. As this study was initiated by two independent projects that later merged, the MIG-seq libraries were adjusted separately at Lake Biwa Museum and Tohoku University (Table 1). However, as we used the same protocol as Suyama, et al.^22^ to adjust the libraries, we considered that the two sequence data sets could be combined. The two libraries were sequenced independently on an Illumina MiSeq platform with a MiSeq Reagent Kit v3 (150 cycles; Illumina, CA, USA). Since we had already sequenced these libraries, the new samples from southern Hokkaido (H4) could not be included in the MIG-seq analysis.

Raw sequences from which primer sequences had already been removed with the “DarkCycle” option of the MiSeq control software were grouped into each index by using the index read option of the sequencer. Quality was filtered with the fastq_quality_filter of the FASTX_Toolkit version 0.0.13 (http://hannonlab.cshl.edu/fastx_toolkit/) with the “-q 30 -p 40” option. Extremely short reads were removed in TagDust version 1.33 with the “-fdr 0.01” option^23^. R1 and R2 reads from the same sample were concatenated into a single fastq file^21^. To remove sequences derived from the organelle genomes, we mapped each read to the mitochondrial and chloroplast genomes of *Vicia fava* KC189947^24^; and *V. sepium* MG682352^25^; in BWA version 0.7.17 with the BWA-MEM algorithm^26^, and unmapped reads were extracted in SAMtools version 1.9^27^.

To obtain maximum and optimum SNPs for each analysis (Table 2), we performed both reference-based and de novo assemblies. For the reference-based assembly, we used the complete genome of *V. sativa* VSA_r1.0^28^. Cleaned reads were mapped to the reference genome with the BWA and BWA-MEM algorithm, and bam files were generated by SAMtools. We then created a catalogue of each assembled locus with the gstacks tool of the Stacks version 2.62. For de novo assembly, we used the ustacks tool with the “-m 3 -M 2” option to assemble each read within an individual. We then created bam files for each individual using the cstacks with the “-n 2” option, sstacks and tsv2bam tools. Finally, we created a catalogue of each assembled locus with gstacks as in the reference-based assembly. In both assemblies, SNPs for each analysis were extracted from bam files and from the catalogue using the populations tool of Stacks with the options shown in Table 2. In addition, pairwise *R*^2^ values for each SNP pair in the datasets for phylogeny, genetic diversity, and population structure analyses were calculated for each SNP pair with PLINK version 1.90 beta 5.2^29^. If *R*^2^ > 0.6, we removed one of the SNPs with a lower genotyping rate to reduce the effect of linkage between SNPs, using the “whitelist” option of the populations tool of Stacks^30^.

**Table 2:** Data set summary for each analysis.

Analysis	Software	Samples	Assembly	Options	Linkage	Number of SNPs	Average genotyping rate (%)
Phylogeny	RAxML-NG	All 85 individuals including outgroups	Reference-based	-R 0.15 --max-obs-het 0.5	Removed one of SNP pairs whose *R*^2^ > 0.6	1725	55.4
Genetic diversity	–	All 83 individuals excluding outgroups	Reference-based	-R 0.15 --max-obs-het 0.5	Removed one of SNP pairs whose *R*^2^ > 0.6	1525	55.4
Population structure	Admixture	77 individuals in 6 Japanese populations	Reference-based	-r 0.30 --min-maf 0.05 --max-obs-het 0.5	Removed one of SNP pairs whose *R*^2^ > 0.6	578	59.7
Past population size change	Stairway plot	67 and 10 individuals in Mt. Ibuki and Central Hokkaido	De novo	-R 0.80 --max-obs-het 0.5	–	747 (Mt. Ibuki)	90.2
						370 (Central Hokkaido)	92.1
Population divergence	fastsimcoal2	67 and 10 individuals in Mt. Ibuki and Central Hokkaido	De novo	-r 0.60 -p 2 --max-obs-het 0.5	–	766	83.7

### Data analysis

Two cpDNA regions were concatenated and a haplotype network was constructed in TCS version 1.21^31^. For the SNP dataset, phylogenetic analysis was performed using a maximum likelihood approach implemented in RAxML-NG version 1.0.3^32^. We used the GTR + Gamma model with an option to correct ascertainment bias (GTGTR4 + G + ASC_LEWIS), and performed 100 bootstrappings. The tree was visualized in FigTree version 1.4.4 (http://tree.bio.ed.ac.uk/software/figtree/).

To compare levels of genetic diversity between Japanese and overseas samples, we calculated the proportion of heterozygous sites within an individual, because the number of overseas samples is small. Differences in the proportion of heterozygous sites between sampled regions, excluding Russia (only one sample), were tested using a generalized linear mixed model with a binomial error distribution implemented in the lme4 package of R version 1.1.31^33^. Individual differences were treated as a random effect (random intercept).

Population structure was estimated with Admixture version 1.3.0^34^. The number of ancestral populations (*K*) was assumed from 1 to 8. The program was run with default settings and performed 10 times for each *K* with a different random seed. To determine the best *K*, we used log-likelihood, *ΔK*^35^, and cross-validation (CV) error. Unlike log-likelihood and *ΔK*, CV-error has the best *K* with the lowest value. We calculated *ΔK* in the corrsieve package of R version 1.6.9^36^. Independent results of the same *K* were merged in CLUMPAK^37^ to draw bar plots of ancestry.

A one-dimensional minor allele site frequency spectrum (1D-mSFS) was calculated from the vcf file with the R script “1D-msfs-R” (https://github.com/garageit46/1D-msfs-R). Missing data were compensated for by bootstrapping. Using the 1D-mSFS, we estimated the trajectory of past effective population size changes with a maximum likelihood method implemented in Stairway plot version 2.1.1^38^. We used a mutation rate of 7 × 10^−9^ per site per generation, which was estimated in *Arabidopsis thaliana*^39^. To convert the time scale from generations to years, we assumed two years per generation, as our study species is a perennial herb.

**Figure 2:**
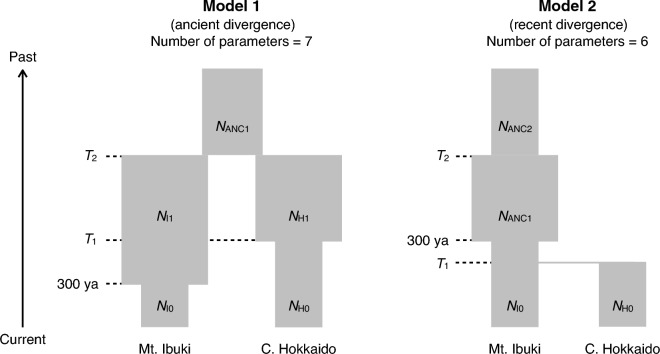
Two divergence models. *N*, effective population size; *T*, event time.

To infer when the Mt. Ibuki and central Hokkaido lineages diverged, we constructed and compared two population divergence models (Fig. 2). Model 1 (ancient divergence) assumes that the two lineages had already diverged before the end of the last glacial period (10^4^ years ago). Model 2 (recent divergence) assumes that they diverged during the colonization of Hokkaido (within the last two centuries) and that the central Hokkaido lineage was introduced from Mt. Ibuki by humans. Any change in population size was assumed from the results of the population size change analysis (see Results). The search ranges of the parameters are summarized in Table S3. We did not consider migration between lineages on account of their separation distance. The same mutation rate and generation time were used as in the population size change analysis. For the observed data, we calculated a two-dimensional minor allele site frequency spectrum (2D-mSFS) from the vcf file with the R script “2D-msfs-R” (https://github.com/garageit46/2D-msfs-R). Missing data were compensated for by bootstrapping within a lineage. The likelihood of each model was maximized from 50 random starting values, 40 expectation-conditional-maximization (ECM) optimization cycles, and 100,000 coalescent simulations in fastsimcoal2 version 2709^40^. We calculated Akaike's information criterion (AIC) and selected the model with the lowest AIC value as the best model. The goodness of fit of the best model was checked by visually comparing the observed and predicted 2D-mSFS. Its confidence interval was calculated by parametric bootstrapping. We simulated the model in fastsimcoal2 with the maximum likelihood estimate parameter values 100 times and obtained its 2D-mSFS. Using the simulated 2D-mSFS as input data, we recalculated the parameters of the best model with the observed parameter values as a starting value, 15 ECM cycles, and 100,000 coalescent simulations. Finally, a 95% confidence interval (CI) was calculated from the obtained parameter values.

We confirm that all the methods were carried out in accordance with relevant Institutional guidelines and regulations.

## Results

### Phylogenetic relationships

In total, we obtained 27,206,661 raw reads after quality filtering and removal of organelle genomic regions from 85 individuals. The average number of raw reads per individual was 320,078 (range 193,678–559,622). These raw reads were used for SNP calling, and we used 370–1725 SNPs in each analysis (Table 2).

**Figure 3:**
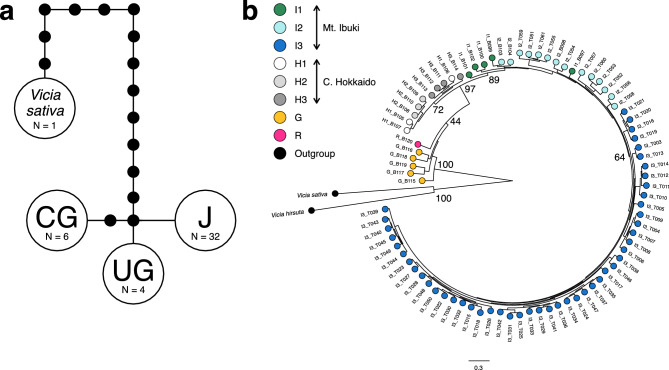
Phylogenetic relationships of *Vicia sepium* samples. Haplotype network of cpDNA sequences (**a**). J, Japanese samples. UG, UK sample and Soest, Germany, samples. CG, Chinese and remaining German samples. ● Missing haplotype. Maximum likelihood phylogenetic tree of nuclear genome (**b**). Numbers indicate bootstrap probability (major nodes only). Abbreviations are as in Table 1.

After alignment of the cpDNA sequences, we obtained 1064 bp concatenated sequences of *rbcL* and *matK*. There were three distinct haplotypes excluding *V. sativa* (Fig. 3a). All 32 Japanese sequences were assigned to haplotype J. All three UK sequences and one from Soest, Germany, were assigned to haplotype UG. Two Chinese sequences and the other four from Germany were assigned to haplotype CG.

On the maximum likelihood phylogenetic tree, *V. sepium* showed clear monophyly with a bootstrap probability of 100% (Fig. 3b). The Japanese samples also showed clear monophyly with a bootstrap probability of 97%. The Mt. Ibuki and central Hokkaido samples showed monophyly, with bootstrap probabilities of 89% and 72%, respectively.

### Genetic diversity and structure

**Figure 4:**
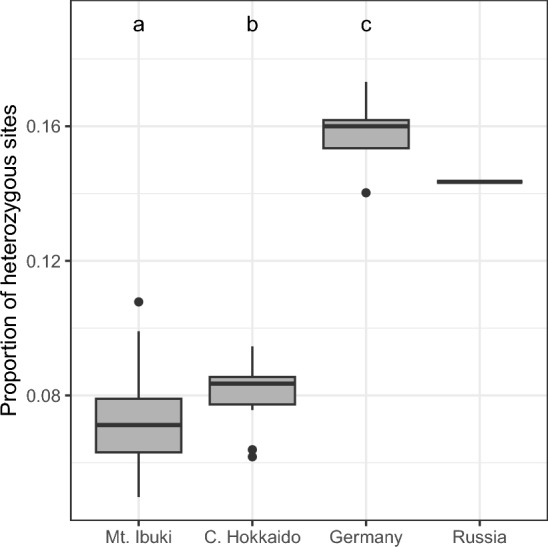
Proportion of heterozygous sites within an individual. Different letters indicate significant differences between regions. The horizontal line indicates the median. Lower and upper limits of the box indicate 25th and 75th percentiles. Lower and upper limits of the vertical bar indicate minimum and maximum values within 1.5 × the length of the box. Dots indicate outliers.

The average proportions of heterozygous sites were 0.071 in Mt. Ibuki samples, 0.081 in central Hokkaido samples, 0.158 in German samples, and 0.143 in Russian samples (Fig. 4). The average values of the proportion of heterozygous sites in the Japanese samples were almost half of that in the overseas samples and significantly lower than that in German samples.

**Figure 5:**
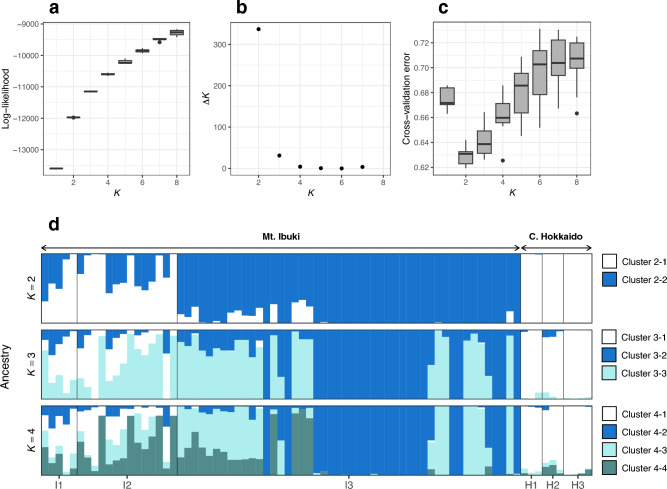
Population structure estimated by Admixture. Changes in (**a**) log-likelihood, (**b**) *ΔK*, and (**c**) cross-validation error with *K*. (**d**) Proportion of ancestries from *K* = 2 to 4.

As the number of ancestral population (*K*) increased, the log-likelihood also increased (Fig. 5a). However, *ΔK* was highest and CV error was lowest at *K* = 2 (Fig. 5b,c). As the CV errors of *K* = 2–4 were also smaller than that of *K* = 1, bar plots of ancestry in *K* = 2–4 are shown (Fig. 5d). At *K* = 2, populations I1 and I2 had a large proportion of cluster 2–1, which was dominant in the central Hokkaido populations (H1–3). However, with increasing *K*, the proportion became smaller and the cluster dominant in the central Hokkaido populations remained dominant only in central Hokkaido.

### Population demography

**Figure 6:**
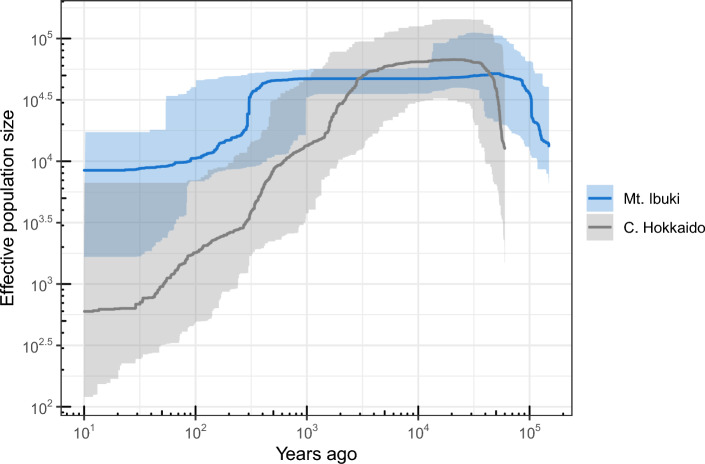
Trajectories of effective population size with time on Mt. Ibuki and in central Hokkaido.

Both lineages showed high effective population sizes during the last glacial period (until 10^4^ years ago; Fig. 6). The central Hokkaido lineage began a gradual decline from 5000 years ago. The Mt. Ibuki lineage maintained a high effective population size until relatively recently, but showed a rapid decrease around 300–400 years ago. Finally, both lineages had a smaller current effective population size than that of the last glacial period.

Model 1 (ancient divergence) had a lower AIC value (ΔAIC = 54.9) than model 2 (recent divergence), and was selected as the best model (Table S4). The divergence time between the lineages was estimated to be 32,792 (95% CI 25,309–38,631) years ago (Table S5).

## Discussion

Although *V. sepium* is widely distributed on the Eurasian continent, its distribution in Japan is very limited. As explained in the introduction, it is possible that each Japanese lineage was introduced in different eras by different pathways. Here we assess this possibility on the basis of the genetic data.

The cpDNA network and nuclear DNA phylogenetic tree showed that the Japanese *V. sepium* lineage was monophyletic and clearly different from overseas samples (German, English, Russian, and Chinese). This indicates that the Mt. Ibuki and Hokkaido lineages have the same origin. Although the cpDNA haplotype of the two lineages was the same, each lineage was monophyletic on the nuclear DNA phylogenetic tree and had a unique ancestry at *K* = 3 and 4 in the genetic structure analysis. Thus, the two Japanese lineages are closely related but clearly distinct. However, from the results of genetic structure analysis, especially at *K* = 2, the genetic cluster dominant in the Hokkaido populations (H1–3) was also admixed in the Mt. Ibuki populations on the Shiga Prefecture side (I1) and the summit of the mountain (I2). Considering these facts, we think it unlikely that each Japanese lineage was introduced in different eras via different routes from the same source. Rather, two rational hypotheses can be considered: (1) Japanese *V. sepium* is not an introduced species but is a native species; and (2) it was introduced from Europe to Mt. Ibuki in the sixteenth century and form there into Hokkaido at the beginning of its colonization. To test these two hypotheses, we made two models. In Model 1 (ancient divergence), the two lineages diverged naturally before the end of the last glacial period, and the observed genetic admixture is due simply to incomplete lineage sorting. In Model 2 (recent divergence), the Hokkaido lineage was introduced recently by humans from Mt. Ibuki during the colonization of Hokkaido (ca. 150 years ago). Model selection supported the ancient divergence model, and the observed genetic admixture was due simply to incomplete lineage sorting. The best model also showed that the two lineages diverged during the last glacial period [maximum likelihood estimate of divergence time was 32,792 (95% CI 25,309–38,631) years ago]. Furthermore, if the Hokkaido lineage was introduced by humans, it should show a sudden population bottleneck in its population-size-change history at that time, because only a very small fraction of genetic diversity would have been introduced^17^. However, the trajectory of its population size change showed a continuous decline. This also rejects the hypothesis that the Hokkaido lineage was introduced by humans from Mt. Ibuki.

On the other hand, the Mt. Ibuki lineage showed a sudden population bottleneck 300–400 years ago. Although this supports the hypothesis that the Mt. Ibuki lineage was introduced by Christian missionaries in the Middle Ages, it contradicts the previous paragraph. Instead, since the whole of Mt. Ibuki has been used by local people from the Middle Ages to about 70 years ago to collect plants for medicine, fertilizer, and feed^6^, it is simpler to assume that this sudden population bottleneck observed is due to such human disturbance. This may become clearer when the population demographies of the other grasses or herbs growing on Mt. Ibuki are investigated in future studies.

All of our population genetic analyses of the SNP data sets generated by MIG-seq are based on the neutral theory. However, since MIG-seq is a genotyping-by-sequencing method closely related to microsatellites, and some microsatellites modulate transcription factors^41^, some SNPs may have been affected by natural selection. In addition, although the recent new evolutionary theory, maximum genetic diversity theory, suggests that the mutation rate changes in different genomic regions depending on the degree of complexity of organisms^42^, our demographic modeling assumes a constant mutation rate throughout the genome, and it may affect the estimation of event time, i.e., divergence time or time for population size change. Therefore, in the future, a more realistic population genetic model will be able to estimate more accurate results.

Although our overseas sample collection is incomplete and sequenced regions of cpDNA are short and limited, even if we will increase overseas samples and sequence more regions, the main result will not change, and therefore, we concluded that the Japanese lineage of *V. sepium* is not introduced but is native. The existence of a recently discovered natural habitat in southern Hokkaido supports this conclusion. Therefore, its existence on Mt. Ibuki does not support the existence of a rumored medicinal plant garden, as mentioned in the introduction. The Mt. Ibuki and the Hokkaido lineages diverged during the last glacial period, and the effective population size of the Hokkaido lineage decreased after the end of the last glacial period, owing to climate change during the Holocene. Although the Mt. Ibuki lineage maintained its effective population size after the end of the last glacial period owing to the endemic environment of the mountain, its effective population size also decreased owing to recent human disturbance. As a result, these two Japanese lineages now have very low genetic diversity compared with overseas *V. sepium* samples. Therefore, the distribution of *V. sepium*, which is widely distributed on the Eurasian continent, is very limited in Japan, perhaps because the Holocene climate of Japan is unsuitable for it. Although there are no plants distributed only in Mt. Ibuki and Hokkaido like *V. sepium*, as the Mt. Ibuki is known to be the southern/western limit of subalpine to alpine plants, for example, *Geranium yesoense*^43^. In addition, Japanese alpine plants distributed from the high mountains of central Japan to Hokkaido have two different origins, southern or northern^44^. Future population genetic studies of *V. sepium* with more detailed sampling in East Asian regions, including the Maritime Province of Siberia surrounding the Sea of Japan may help to elucidate its origin.

### Supplementary Information

Supplementary Tables.

## Data Availability

The cpDNA sequence and genome wide SNP datasets generated and/or analyzed during the current study are available in the DDBJ/EMBL/GenBank and Dryad repository (Accession # LC767570–LC767640 and LC769859–LC769864; and 10.5061/dryad.63xsj3v7t, respectively).
