# Supplementary material for: Phylogenetic, population structure, and population demographic analyses reveal that Vicia sepium in Japan is native and not introduced
Source: Sci Rep. 2023 Nov 25;13:20746. doi: 10.1038/s41598-023-48079-4 (PMC10676423; doi:10.1038/s41598-023-48079-4)
Supplement: Supplementary file 1 — Supplementary Tables. [file 41598_2023_48079_MOESM1_ESM.docx]

Online support materials

*Scientific Reports*

Phylogenetic, population structure, and population demographic analyses reveal that *Vicia sepium* in Japan is native and not introduced

Ichiro Tamaki ^1, 2^, Mizuo Mizuno ^3^, Tatsuo Ohtsuki ^4^, Kohtaroh Shutoh ^5^, Ryoichi Tabata ^4^, Yoshihiro Tsunamoto ^6, 7^, Yoshihisa Suyama ^7^, Yusuke Nakajima ^8^, Naoki Kubo ^8^, Takeru Ito ^8^, Naohiko Noma ^8^, Emiko Harada ^8^*

^1^ Gifu Academy of Forest Science and Culture, 88 Sodai, Mino, Gifu 501-3714, Japan

^2^ Gifu Prefectural Research Institute for Forests, 1128-1 Sodai, Mino, Gifu 501-3714, Japan

^3^ Gifu Pharmaceutical University, 5-6-1 Mitahora-higashi, Gifu 502-8585, Japan

^4^ Lake Biwa Museum, 1091 Oroshimo, Kusatsu, Shiga 525-0001, Japan.

^5^ The Hokkaido University Museum, Kita 10, Nishi 8, Kita-ku, Sapporo, Hokkaido 060-0810, Japan

^6^ Research Institute of Energy, Environment and Geology, Hokkaido Research Organization, Kita 19 Nishi 12, Kita-ku, Sapporo, Hokkaido, 060-0819 Japan

^7^ Kawatabi Field Science Center, Graduate School of Agricultural Science, Tohoku University, 232-3 Yomogida, Naruko-onsen, Osaki, Miyagi 989-6711, Japan

^8^ School of Environmental Science, The University of Shiga Prefecture, 2500 Hassaka-cho, Hikone City, Shiga 522-8533, Japan

*Corresponding author: Emiko Harada

Email: harada.e@ses.usp.ac.jp

**Table S1.** Primers used for amplification of *rbcL* and *matK* regions in cpDNA

| Region | F/R | Sequence 5'–3' | Reference |
| --- | --- | --- | --- |
| *rbcL* | F | ATGTCACCACAAACAGAGACTAAAGC | Kress and Erickson (2007) |
|  | R | GTAAAATCAAGTCCACCRCG | Kress et al. (2009) |
| *matK* | F | CTTATAAAAATTTGCGATCAATTCATTC | de Vere et al. 2012 |
|  | R | CTCTTCTTCCTCTGTAAAGAATTCT | de Vere et al. 2012 |

de Vere N, Rich TCG, Ford CR, Trinder SA, Long C, Moore CW, Satterthwaite D, Davies H, Allainguillaume J, Ronca S, Tatarinova T, Garbett H, Walker K, Wilkinson MJ (2012) DNA barcoding the native flowering plants and conifers of Wales. PLoS ONE 7:e37945

Kress WJ, Erickson DL (2007) A two-locus global DNA barcode for land plants: The coding *rbcL* gene complements the non-coding *trnH*-*psbA* spacer region. PLoS ONE 2:e508

Kress WJ, Erickson DL, Jones FA, Swenson NG, Perez R, Sanjur O, Bermingham E (2009) Plant DNA barcodes and a community phylogeny of a tropical forest dynamics plot in Panama. Proceedings of the National Academy of Sciences 106:18621–18626

**Table S2.** Used cpDNA sequences obtained from GenBank

| Accession No. | |  |  |
| --- | --- | --- | --- |
| *rbcL* | *matK* | Species | Sample site |
| JN893678 | JN896140 | *Vicia sepium* | Glamorgan, UK |
| JN891169 | JN894332 | *Vicia sepium* | Glamorgan, UK |
| JN893681 | JN896142 | *Vicia sepium* | Cardiganshire, UK |
| MG682352 | MG682352 | *Vicia sepium* | Hunan, China |
| NC_039595 | NC_039595 | *Vicia sepium* | Hunan, China |
| NC_027155 | NC_027155 | *Vicia sativa* | USDA germplasm collection at Pullman, Washington (Seed accession ID: 293436) |

**Table S3.** Search range of parameters of population divergence models

| Model | Parameter | Distribution | Lower | Upper |
| --- | --- | --- | --- | --- |
| 1 | *N*_I0_ | Log-uniform | 10 | 1×10^5^ |
|  | *N*_H0_ | Log-uniform | 10 | 1×10^5^ |
|  | *N*_I1_ | Log-uniform | *N*_I0_ | 1×10^5^ |
|  | *N*_H1_ | Log-uniform | *N*_H0_ | 1×10^5^ |
|  | *N*_ANC_ | Log-uniform | 10 | 1×10^6^ |
|  | *T*_1_^a^ | Log-uniform | 150 | 5000 |
|  | *T*_2_^a^ | Log-uniform | 5000 | 1×10^5^ |
| 2 | *N*_I0_ | Log-uniform | 10 | 1×10^5^ |
|  | *N*_H0_ | Log-uniform | 10 | 1×10^5^ |
|  | *N*_ANC1_ | Log-uniform | *N*_I0_ | 1×10^6^ |
|  | *N*_ANC2_ | Log-uniform | 10 | *N*_ANC1_ |
|  | *T*_1_^a^ | Uniform | 50 | 100 |
|  | *T*_2_^a^ | Log-uniform | 150 | 1×10^5^ |

^a^ Unit of time parameters are generations ago. Two years per generation was assumed.

**Table S4.** Parameters of compared two population divergence models

| Parameter | **Model 1** | Model 2 |
| --- | --- | --- |
| NP | **7** | 6 |
| LL | **-3703** | -3715 |
| AIC | **17067** | 17122 |
| *N*_I0_ | **5708** | 555 |
| *N*_H0_ | **936** | 151 |
| *N*_I1_ | **42161** | – |
| *N*_H1_ | **64248** | – |
| *N*_ANC1_ | **35254** | 860902 |
| *N*_ANC2_ | **–** | 18733 |
| *T*_1_ (ya) | **692** | 196 |
| *T*_2_ (ya) | **32792** | 95622 |

NP, number of parameters; LL, log-likelihood in log_10_ scale; AIC, Akaike's information criterion; *N*, effective population size; *T*, event time.

The best model was shown in bold.

**Table S5.** Maximum likelihood estimate (95 % confidence interval) of parameters in the best model (model 1)

| Parameter | MLE (95 % CI) |
| --- | --- |
| *N*_I0_ | 5708 (3786–11454) |
| *N*_H0_ | 936 (714–1930) |
| *N*_I1_ | 42161 (33767–52177) |
| *N*_H1_ | 64248 (26910–80275) |
| *N*_ANC1_ | 35254 (31515–39510) |
| *T*_1_ (ya) | 692 (421–1014) |
| *T*_2_ (ya) | 32792 (25309–38631) |

*N*, effective population size; *T*, event time.
